# Research trends on endoscopic mucosal resection: A bibliometric analysis from 1991 to 2021

**DOI:** 10.3389/fsurg.2022.994718

**Published:** 2022-10-20

**Authors:** Yihan Yang, Xuan Xu, Menghui Wang, Yang Zhang, Pinglang Zhou, Sifan Yang, Xu Shu, Chuan Xie

**Affiliations:** ^1^ Department of Gastroenterology, Digestive Disease Hospital, The First Affiliated Hospital of Nanchang University, Nanchang, China; ^2^ Faculty of Basic Medical Sciences, Xizang Minzu University, Xianyang, China

**Keywords:** bibliometric analysis, publications, research trends, surgical procedure, endoscopic mucosal resection

## Abstract

**Background:**

In recent years, the rapid development of digestive endoscopy technology has brought revolutionary changes to endoscopic therapy. A growing number of articles have been published annually. We aimed to explore global scientific outputs and hotspots of endoscopic mucosal resection (EMR) published by different countries, organizations, and authors.

**Methods:**

We extracted relevant publications from the Web of Science Core Collection (WOSCC) on June 23, 2022. We examined the retrieved data by bibliometric analysis (e.g., cocited and cluster analysis, keyword co-occurrence) using the software CiteSpace and VOSviewer to analyze and predict the trends and hot spots in this field.

**Results:**

A total of 2,695 papers were finally identified. The results showed that the number of articles fluctuated with the year and reached its peak in 2021. NATIONAL CANCER CENTER JAPAN was the most influential institution. MICHAEL J BOURKE and YUTAKA SAITO are two of the most prolific scholars. ENDOSCOPY and GASTROINTESTINAL ENDOSCOPY were the most productive journals. “Early gastric cancer” and “Barrett's esophagus” were the focus of EMR research. “Adverse events”, “cold snare polypectomy” and “outcomes” have become increasingly popular in recent years and could become hot spots in the future.

**Conclusion:**

In this study, we summarized the characteristics of the publications; identified the most influential countries, institutions, and journals; and identified the leading topics in the EMR field.

## Introduction

Endoscopic mucosal resection (EMR) is an endoscopic treatment technique pioneered in Japan by Soetikno et al. (1). Initially, EMR was mainly used for the treatment of early gastric cancer, but it is now widely used for the treatment of superficial lesions of gastrointestinal mucosa larger than 2 cm, such as small intestinal adenoma or colorectal adenoma (2–7). After nearly 50 years of development, endoscopic mucosal resection has gradually matured, and new relevant procedural methods have been derived (4, 8–10). Furthermore, endoscopic submucosal dissection (ESD) has been derived from EMR, but it has a unique theory and operating system. EMR and ESD procedures have both become common in their relevant fields.

Bibliometric analysis is an important method used to identify the research focus and trend of a certain field (11). This method first obtains the data of a field from the literature database, makes a quantitative analysis of the extracted data, then obtains the literature quality and academic influence of this field, and finally identifies the future development direction of a particular field (12–14). WOS, the largest comprehensive academic information database in the world, is used in this study to ensure the data accuracy of the bibliometric analysis (15). In addition, to make the quantitative analysis results more intuitive, we also used VOSviewer and CiteSpace to perform a visual analysis of the retrieval results (15, 16).

At present, some studies have reported endoscopic treatment techniques using bibliometric analysis, but there is no bibliometric analysis of endoscopic mucosal resection (17). Therefore, this study aims to analyze the research hotspots and trends in this field from 1991 to 2021 by using quantitative literature analysis to fill in the research gaps and provide assistance for the research and clinical application of endoscopic mucosal resection.

## Materials and methods

### Search strategy

A comprehensive literature search was performed in the WOS Core database (Clarivate Analytics, United States), which is considered the most appropriate database for bibliometric analysis. We used WOS to identify the most frequently cited papers. The reason for using this database is that it is the most frequently used and accepted database in scientific or bibliometric research, includes almost all influential and high-quality journals, and contains a comprehensive citation index record. We searched relevant kinds of literature involving “endoscopic mucosal resection” or “endoscopic mucosal resections” or “endoscopic mucous membrane resection” or “mucosal resection and endoscopic” or “resection and endoscopic mucosal” in their titles or keywords.

### Study selection

Two authors (YYH and XX) independently confirmed all relevant publications, including the title, keywords, publication year, country/region, institution, author, and citation count. We applied filters to limit the search to original articles and reviews. The period of the literature search was from 1991 to 2021, and non-English literature was excluded from our study. We completed our literature retrieval and data downloads in the course of 1 day, June 23, 2022, to reduce bias arising from frequent updates of the database. Given that data were directly downloaded from the database, ethical approval was not needed.

### Data extraction and analysis

For bibliometric analysis, we first converted the WOSCC data to TXT format. Next, we imported these data into CiteSpace V5.7.R3 SE, 64-bit (Drexel University, Philadelphia, USA) and VOSviewer1.6.15 (Leiden University, Netherlands). Then, we performed quantitative and qualitative analyses of these data in CiteSpace and VOSviewer.

In CiteSpace, we used the following options: the time slice is set to “1991–2021”, the number of years for each slice is set to “1”, the selection criteria are set to “g-index”, and the scale factor k is set to “25”. Moreover, to preserve the most significant structure and reduce the number of links, we selected the options “pathfinder” and “trim the merged network”. For node types, we could only select one option at a time from “author”, “institution”, “country”, “reference”, “citation author” and “keywords” (18).

The role of CiteSpace is important for bibliometric analysis, which can explore the knowledge base and frontier of a certain research field by performing cocitation analysis and burst detection. When two articles are simultaneously cited by a third article, the two articles form a cocitation relationship. The strength of the cocitation relationship between two cited papers is directly proportional to the similarity of their research content. The more times they are cited at the same time, the stronger the cocitation relationship. In addition, references with a strong cocitation relationship form a certain category reflecting the same research topic. Centrality is a major indicator to determine the importance of nodes in the network and a higher centrality means that the node is more important in this network. Generally, nodes with a Centrality value of more than 0.1 occupy pivotal positions connecting a large number of nodes and are usually identified as hubs of nodes displayed in purple.

We selected the top 25 keywords with the largest citation volume to explore the research hotspots of EMR. In the burst detection results, “Begin” refers to the year when a burst of citation begins with a reference or keyword, and “End” refers to the year when a burst of citation ends with a reference or keyword. The red line refers to the duration of the burst of citation, and “Strength” refers to the intensity of the burst of citation.

In VOSviewer, we created keyword maps by using the following options: “create a map based on bibliographic data”, “read data from bibliographic database files”, “analysis type: co-occurrence”, “analysis unit: all keywords”, “count method: full count”, and “minimum occurrences of keywords: 20”. The circle size of a project is proportional to its number of publications. The width of the lines between the two projects is proportional to the level of cooperation. Objects of the same color belong to the same cluster, which indicates that they cooperated closely in this field. Meanwhile, the journal impact factors and quartiles of the journal category were identified according to the journal citation report 2021 criteria to evaluate the scientific impact of the country/region and journal (https://jcr.clarivate.com/).

## Results

### Study literature selection

**Figure 1:**
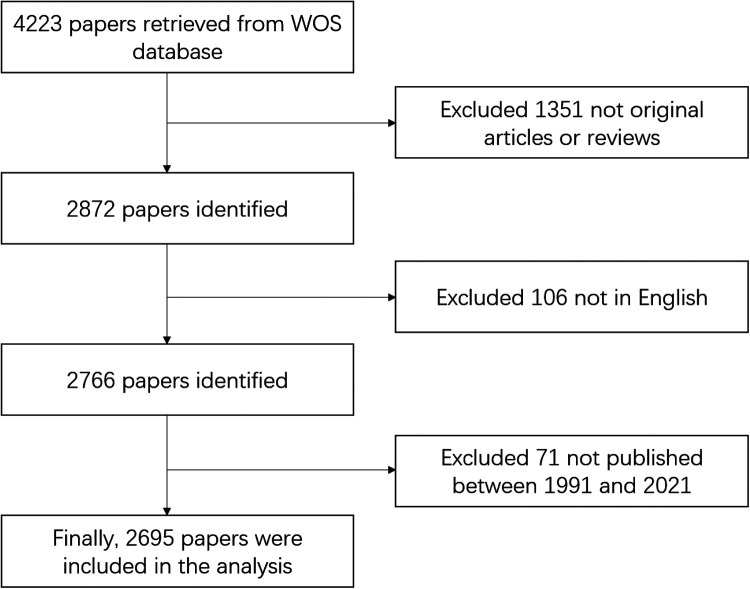
Flow chart of the literature screening process.

We obtained 4,223 papers from WOSCC by searching for relevant keywords concerning EMR. Excluding nonoriginal articles and reviews and non-English language papers, 2,766 papers were obtained. Then, excluding papers not published between 1991 and 2021, we finally obtained 2,695 papers to analyze in this study. The flow chart for this retrieval is shown in Figure 1.

**Figure 2:**
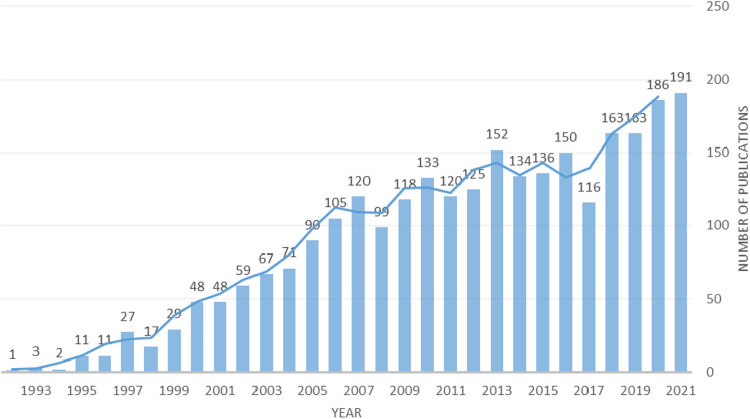
Chronological distribution of the publications from 1991 to 2021.

After screening, a total of 2,695 publications were identified in the present study, including 2,266 (84.1%) original articles and 429 (15.9%) reviews. Figure 2 shows the chronological distribution of the publications from 1991 to 2021. The first article we retrieved was published in 1991. INOUE, H reported a case of carcinoma *in situ* of the esophagus accompanied by esophageal varices that were treated by endoscopic mucosal resection using a transparent tube following eradication of the varices *via* injection sclerotherapy. As depicted in the diagram, the number of articles and reviews grew steadily in the past two decades and peaked in 2021.

### Analysis of institution and country

**Figure 3:**
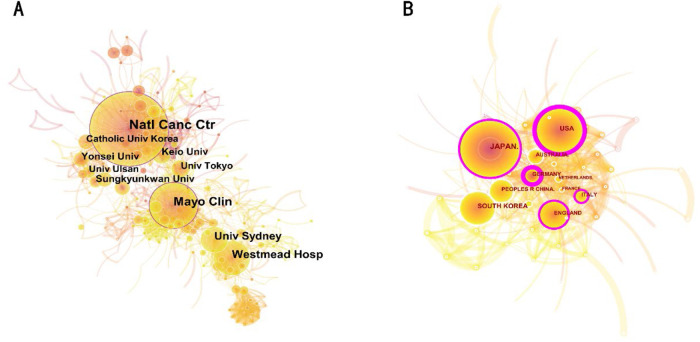
(**A**) The co-occurrence map based on countries. (**B**) The co-occurrence map is based on institutions.

At least 2,349 institutions from 65 different countries/regions have published papers on EMR. The papers are mainly on Japan (958 papers, 35.5%) and the United States (606 papers, 22.5%), and they have together published more than half of the relevant studies on EMR. South Korea (261 papers, 9.7%), China (205 papers, 7.6%), and the United Kingdom (176 papers, 6.5%) also contributed significantly to EMR research. Germany, the United States, Japan, the United Kingdom, and Italy scored highest for the centrality of research cooperation. Table 1 shows the detailed distribution of these countries/institutions, and Figure 3A shows their co-occurrence networks. According to Table 2, five of the top 10 research institutions with the most publications are based in South Korea, two are in Japan, two in Australia, and one in the United States. Table 3 shows the top 10 most cited institutions, none of which are from South Korea. The organization with the most publications is the NATIONAL CANCER CENTER JAPAN. The symbiotic network between research institutions shows a low-density graph (density = 0.0091), with most of the centrality below 0.1 in Figure 3B. This means that research groups are relatively dispersed across institutions, but most institutions have a restrictive influence in the field.

**Table 1:** Top 10 countries in terms of publications.

Rank	Country/region	Records	Centrality	Total number of citations
1	JAPAN	958	0.15	37,347
2	USA	606	0.21	19,027
3	SOUTH KOREA	261	0.02	5,674
4	PEOPLES R CHINA	205	0.03	2,640
5	ENGLAND	176	0.19	7,781
6	GERMANY	149	0.25	8,503
7	AUSTRALIA	116	0.05	5,091
8	ITALY	111	0.14	4,278
9	NETHERLANDS	84	0.03	4,728
10	FRANCE	68	0.06	2,988

**Table 2:** Top 10 institutions in terms of publications.

Rank	Institutions	Records	Centrality	Total number of citations
1	NATIONAL CANCER CENTER JAPAN	126	0.19	9,825
2	MAYO CLIN	75	0.11	3,438
3	WESTMEAD HOSPITAL	66	0.01	3,319
4	UNIVERSITY OF SYDNEY	53	0.02	2,756
5	SUNGKYUNKWAN UNIVERSITY SKKU	41	0.01	1,015
6	UNIVERSITY OF TOKYO	39	0.03	1,413
7	YONSEI UNIVERSITY	38	0.00	1,071
8	KEIO UNIVERSITY	37	0.06	935
9	CATHOLIC UNIVERSITY OF KOREA	36	0.02	477
10	UNIVERSITY OF ULSAN	34	0.01	516

**Table 3:** Top 10 institutions in terms of citations.

Rank	Organization	Documents	Citations
1	NATIONAL CANCER CENTER JAPAN	126	9,825
2	MAYO CLIN	75	3,438
3	WESTMEAD HOSPITAL	66	3,319
4	UNIVERSITY OF SYDNEY	53	2,756
5	NATIONAL CANCER CENTER HOSPITAL EAST	29	2,312
6	THE UNIVERSITY OF NORTH CAROLINA	29	2,266
7	SHOWA UNIVERSITY	27	2,174
8	STANFORD UNIVERSITY	30	1,988
9	KLINIKUM BAYREUTH	12	1,964
10	QUEEN ALEXANDRA HOSPITAL	19	1,925

### Analysis of authors

**Figure 4:**
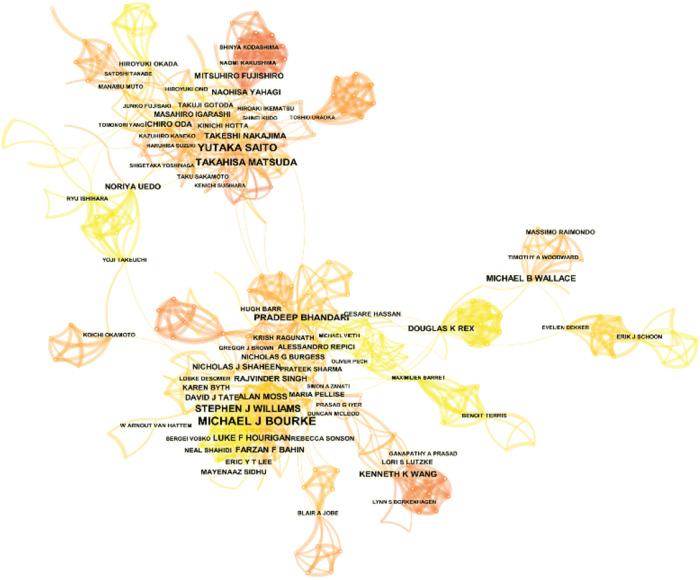
Co-occurrence map based on authors.

A total of 12,052 authors participated in these papers, 106 of whom published more than 10 studies. Table 4 shows the 10 most prolific authors in the study. Bourke, Michael J. from the Department of Gastroenterology of WESTMEAD HOSPITAL in Sydney, Australia ranked first in the number of papers published (63 papers). This was followed by YUTAKA SAITO (50 papers) and TAKAHISA MATSUDA (33 papers) of the Department of Endoscopy, National Cancer Center, Japan. It is worth noting that the number of cited papers of MICHAEL J BOURKE and YUTAKA SAITO is significantly higher than all researchers, indicating that the two authors have made great achievements and have become authorities in the field of EMR research. We visualized the authors using CiteSpace software in Figure 4. The network among authors showed a low density (density = 0.0054). These authors are groups, but there is a lack of cooperation between the groups.

**Table 4:** Top 11 authors in terms of publications.

Rank	Authors	Records	Total number of citations
1	MICHAEL J BOURKE	63	2,834
2	YUTAKA SAITO	50	2,999
3	TAKAHISA MATSUDA	33	1,812
4	STEPHEN J WILLIAMS	31	2,286
5	NAOHISA YAHAGI	25	1,385
6	RAJVINDER SINGH	24	1,627
7	MICHAEL B WALLACE	23	1,467
8	KENNETH K WANG	23	877
9	PRADEEP BHANDARI	23	1,475
10	ICHIRO ODA	22	1,556
11	TAKESHI NAKAJIMA	22	1,295

### Analysis of journals

**Figure 5:**
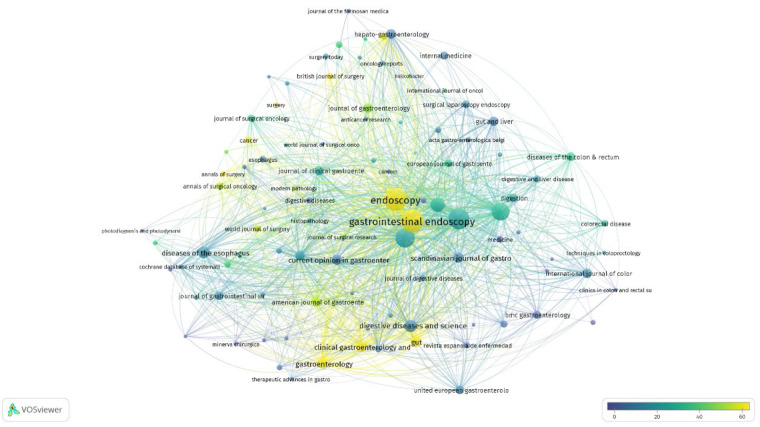
Co-occurrence map based on journals.

A total of 365 journals published papers in the EMR field. Among 2,695 papers concerning EMR in our study, 1,086 papers (40.3%) were published in the top 11 journals, as shown in Table 5. Judging by the number of posts, the top 3 were ENDOSCOPY (IF = 9.776), GASTROINTESTINAL ENDOSCOPY (IF = 10.396), and SURGICAL ENDOSCOPY AND OTHER INTERVENTIONAL TECHNIQUES (IF = 3.453). It is worth noting that GASTROENTEROLOGY (IF = 33.883) far outperformed the other journals in terms of the number of average citations. The top 3 most published journals in the field of EMR are endoscopy journals. These journals have shown a strong interest in EMR and are more likely to accept articles in this area. The coauthorship analysis of journals was performed, and a network map was constructed, as shown in Figure 5. The weight of the node is represented by the number of posts, and the color of the node is represented by the average number of citations.

**Table 5:** Top 11 journals in terms of publications.

Rank	Journals	Records	Total number of citations	Average number of citations	IF2021	JCR
1	ENDOSCOPY	189	11,175	59.1	9.776	Q1
2	GASTROINTESTINAL ENDOSCOPY	187	11,770	62.9	10.396	Q1
3	SURGICAL ENDOSCOPY AND OTHER INTERVENTIONAL TECHNIQUES	156	3,693	23.7	3.453	Q1
4	WORLD JOURNAL OF GASTROENTEROLOGY	137	2,967	21.7	5.374	Q2
5	DIGESTIVE ENDOSCOPY	118	3,450	29.2	6.337	Q1
6	JOURNAL OF GASTROENTEROLOGY AND HEPATOLOGY	73	2,024	27.7	4.369	Q2
7	DIGESTIVE DISEASES AND SCIENCES	55	650	11.8	3.487	Q2
8	DISEASES OF THE ESOPHAGUS	50	786	15.7	2.822	Q3
9	CLINICAL GASTROENTEROLOGY AND HEPATOLOGY	41	2,811	68.6	13.576	Q1
10	GASTROENTEROLOGY	40	4,653	116.3	33.883	Q1
11	SCANDINAVIAN JOURNAL OF GASTROENTEROLOGY	40	488	12.2	3.027	Q3

### Analysis of keywords and burst detection

Keyword co-occurrence analysis provides a detailed description of hot topics involved in EMR research. Each paper has corresponding keywords. By analyzing the titles and abstracts of the included papers, VOSviewer identified 183 keywords that appeared at least 20 times and visualized citation data with a bubble graph. In the VOSviewer keyword co-occurrence visualization map, all keywords are grouped into clusters, and different clusters are marked by different colors.

**Figure 6:**
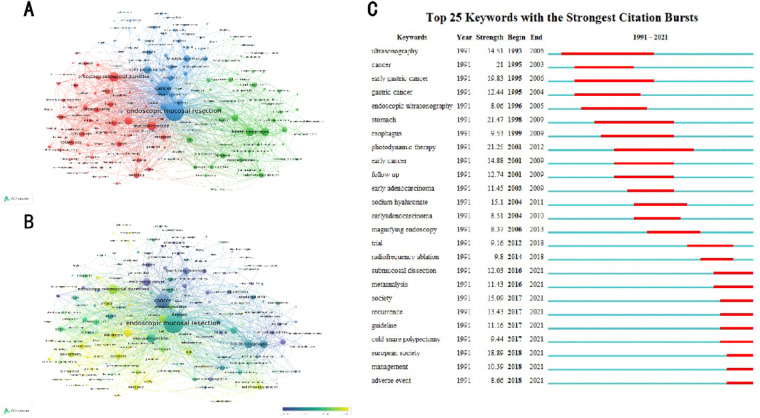
(**A,B**) The co-occurrence map based on keywords. (**C**) The top 25 keywords with the strongest citation bursts.

The three categories are “early gastric cancer”, “colorectal cancer”, and “Barrett's esophagus” in Figure 6A. There is a color bar in the lower right corner of the map with keywords in different colors based on the average year of publications in Figure 6B. For example, “early gastric cancer” and “lymph node metastasis” mainly appeared before 2010. In subsequent years, the keywords “Barrett's esophagus”, “endoscopic submucosal dissection”, “colorectal cancer” were more common. Keywords marked in yellow, such as “outcomes,” “safety,” “colonoscopy,” “adverse events,” and “cold snare polypectomy” are the latest. This shows that these areas have become increasingly popular in recent years and could become hot spots in the future.

Burst detection reveals a sudden increase in emerging concepts over time. The timeline is drawn as a blue line divided into years. The periods in which a topic's outbreak was observed to be marked in red indicate the start and end years and duration of the referenced outbreak. The burst pattern of keywords reveals new content and the relevant research focus in EMR. We combined keywords of the same meaning, such as carcinoma, tumors, and cancer. Figure 6C shows that, over the past three decades, the stomach ranked first in terms of outbreak intensity (21.47), followed by photodynamic therapy (21.25), cancer (21), and early gastric cancer (19.83). Since 1993, endoscopic ultrasonography, cancer, and early gastric cancer have become the focus of research, followed by lining and photodynamic therapy. It is worth noting that adverse events, management, European society, cold snare polypectomy, and recurrence were the strongest bursts since 2017, indicating that they have become new research topics of EMR.

### Cocited reference cluster analysis

**Figure 7:**
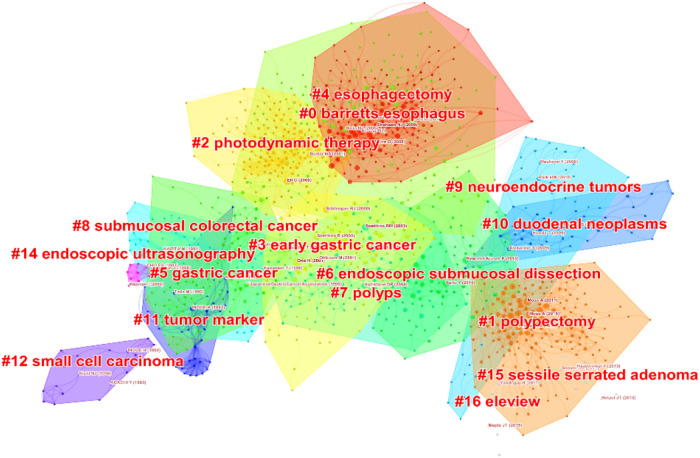
The cluster network diagrams.

**Figure 8:**
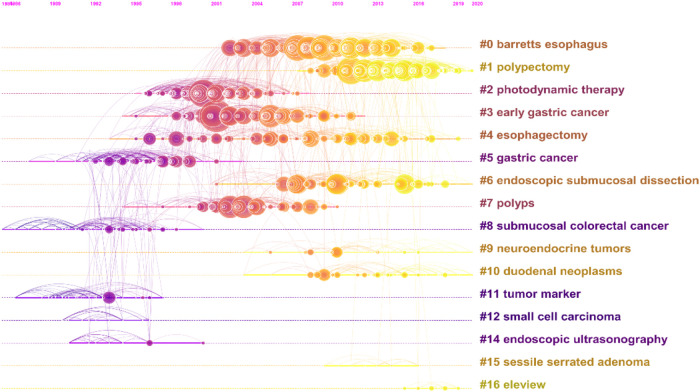
The literature cocitation diagrams.

A cocited network is a network of references cocited by some group of publications. A concept cluster is an edge generated when a group of references is repeatedly cited. We generated cluster network diagrams (Figure 7) and literature cocitation diagrams (Figure 8) through CiteSpace. We chose the “Pathfinder” and “Pruning Networks” options to preserve the best network structure. The visualization with references shows 1,455 nodes and 3,844 links. In this network, each node represents a cited article, and the size of each node is proportional to the total cited frequency of relevant articles. In Figure 7, the cocited literature is grouped into 16 major classification labels, indicating that the field of endoscopic mucosal resection has focused on the following 16 topics in the past 30 years: Barrett's esophagus, polypectomy, photodynamic therapy, early gastric cancer, esophagectomy, gastric cancer, endoscopic submucosal dissection, polyps, colonic lesions invading the submucosa, neuroendocrine tumors, duodenal neoplasms, tumor marker, small cell carcinoma, endoscopic ultrasonography, sessile serrated adenoma and eleview. The smaller the label number, the more literature it contains. We list the information for each cluster in Table 6. The silhouette value of a cluster reflected its homogeneity. The closer the value was to 1, the more homogenous it was. When the silhouette value of a cluster was >0.7, this cluster could be considered highly reliable. The silhouette of the 16 clusters ranged from 0.867 to 1, reflecting their relatively high homogeneity. A timeline view of distinct cocitations is presented in Figure 8. According to Figure 8, the research hotspot and topics have shifted from gastric cancer, colonic lesions invading the submucosa, tumor marker and endoscopic ultrasonography to Barrett's esophagus, polypectomy, endoscopic submucosal dissection, neuroendocrine tumors, duodenal neoplasms and esophagectomy.

**Table 6:** The cluster details.

Cluster ID	Size	Silhouette	Mean (Year)	Label (LLR)
0	198	0.913	2009	Barrett's esophagus
1	178	0.959	2014	Polypectomy
2	158	0.952	2001	Photodynamic therapy
3	125	0.924	2003	Early gastric cancer
4	115	0.893	2008	Esophagectomy
5	113	0.867	1995	Gastric cancer
6	93	0.908	2010	Endoscopic submucosal dissection
7	85	0.932	2003	Polyps
8	61	0.948	1992	Colonic lesions invading the submucosa
9	44	0.996	2012	Neuroendocrine tumors
10	40	0.981	2013	Duodenal neoplasms
11	40	0.922	1991	Tumor marker
12	20	0.988	1992	Small cell carcinoma
14	10	0.996	1993	Endoscopic ultrasonography
15	9	0.994	2012	Sessile serrated adenoma
16	8	1	2017	Eleview

## Discussion

This study focused on the field of EMR. After searching the WOSCC and manually screening eligible studies, a total of 2,695 studies were analyzed. We analyzed the annual number of publications concerning EMR from 1993 to 2021, finding that the number peaked at 120 in 2007 and was followed by a steady increase. It was surprising that the number of publications reached 186 in 2019 and 191 in 2021. According to the data, the number of publications reached 186 and 191, respectively, indicating that EMR has become an important field of relevant endoscopy research in recent years and that this field has entered a relatively stable publishing stage. Next, we analyzed the most influential countries, institutions, and journals in the field of EMR. Among these countries, JAPAN ranked first in the number of records and total number of citations, and the USA ranked second. The USA ranked first in centrality, and JAPAN came in second. Moreover, JAPAN and the USA are the centers of the country co-occurrence map, indicating that JAPAN and the USA are the most influential countries in the EMR field. In the institutional analysis, we found that the NATIONAL CANCER CENTER JAPAN was the most productive institution, with the highest number of records (126), centrality (0.19), and total number of citations (9,825), was present in the center of the network and was closely connected with other institutions. The NATIONAL CANCER CENTER is the most influential institution in this field. In the author analysis, MICHAEL J BOURKE and YUTAKA SAITO have a large number of citations. Bourke, Michael J. had a significant record of achievement, with the highest citation frequency of 449 (19). YUTAKA SAITO also made a great contribution in this field, with the highest citation frequency of 556 (20). This means that they are both authoritative researchers and experts in this field. In the journal analysis, we found that the 11 most productive journals with high 2021 journal IF. ENDOSCOPY and GASTROINTESTINAL ENDOSCOPY, with the highest number of publications and total number of citations, respectively.

Then, we analyzed the keywords of the EMR field from 1991 to 2021. Based on keyword co-occurrence analysis, we reviewed and analyzed the history and some research achievements of EMR. Initially, people only used it to treat early gastric cancer, which is why “early gastric cancer” was a hot keyword in the field before 2000 (1, 21, 22). In the following years, studies found that EMR also achieved good results in the treatment of esophageal tumors or colorectal adenomas, so keywords such as “Barrett's esophagus” and “colorectal cancer” also emerged in large numbers (23–27). Since 2012, “neuroendocrine tumor” and “duodenal adenoma” have become research hotspots in the field of EMR. Because EMR has developed to date, it is not limited to traditional EMR procedures but also involves cap-assisted (EMR-C), underwater endoscopic mucosal resection (U-EMR) and other new technologies (28, 29). EMR-C can completely remove neuroendocrine tumors with no signs of recurrence for 24 months after treatment (30). U-EMR, first reported by Binmoeller in 2013, can be used to remove duodenal adenomas more efficiently (31). These new technologies have strongly promoted the development of EMR in clinical treatment. In addition, keyword co-occurrence analysis further found that since 2018, terms such as “adverse events” and “safety” have appeared in the literature in large numbers. This indicates that while improving the removal rate of tumors or polyps by EMR, researchers also are also concerned with the adverse events caused by EMR, such as delayed bleeding and perforation (32, 33). Takayuki et al. conducted a single-center retrospective trial to investigate the resectability of underwater endoscopic mucosal resection for duodenal tumor. The results showed that U-EMR may have the potential to be the first choice for small to medium-sized NADET (34). Recent studies have also demonstrated complete resection of anterior pyloric intramucosal gastric cancer by underwater endoscopic mucosal resection (35). Meanwhile, Sung Kyu Choi et al. reported the results of U-EMR in benign mucosal tumors located in the pyloric ring (36). Concern about complications is also one of the important characteristics of EMR technology that is maturing.

From the quantitative analysis results, we found that EMR continues to improve in injection reagents, ligation methods and other aspects. In terms of injection reagents, a new submucosal injection “eleview” has emerged. After the birth of eleview in 2017, there have been an increasing number of reports about eleview injections. Compared with traditional normal saline, eleview injections can improve the overall tumor resection rate with less dosage (37–39). A recent comparative trial found that eleview injections significantly increased the height and duration of mucosal lift compared with saline injections, creating a buffer layer for polyp removal surgery and improving the success rate of surgery. Therefore, eleview is expected to be an effective alternative to saline injection (40). The keyword “cold snare polypectomy” exploded in 2017, and the use of cold snares to ligate tumors or polyps is becoming a popular research topic. Cold snare polypectomy eliminates the need for traditional EMR electrocautery, achieving a near 100% cure rate for large (≥20 mm) sessile serrated lesions (L-SSL), and minimizes unscheduled hospital admission time (41, 42). In addition, other studies have found that the use of cold snare polypectomy can effectively avoid heat injury and reduce the incidence of perforation and bleeding, two common complications in conventional EMR (42–44). Therefore, cold-snap-induced resection may become the future development direction of EMR, and EMR and its derivative technology will be applied in more fields. However, a large sample size and prospective experiments and data analysis are still needed to further develop EMR.

Despite the contributions of this study, there are some inevitable limitations. First, we only searched for publications using WOSCC, which may lead to incomplete literature searches of other databases, and this bias may affect the final results. The Web of Science Core Collection is the most widely used database in bibliometric analyses and was built for this type of analysis. Second, we may have missed a number of studies that were not in English. For example, Japan has many influential scholars in the EMR field who may publish in Japanese, but non-English literature makes up a small percentage of the literature we targeted for this study, so it had little to no effect on the final results. Finally, some biases may affect the results, such as publication bias. Regarding publication bias, positive results are more likely to be published, but the degree of impact on our current study is minimal.

In conclusion, in this study, we used various statistical software programs and analyzed different extracted data to gain an overview of the field of EMR. We examined the publication characteristics; identified the most influential countries, institutions, and journals; and identified the research hotspots and trends in the EMR field.

## Data Availability

The original contributions presented in the study are included in the article/Supplementary Material, further inquiries can be directed to the corresponding author/s.
